# The Potential of Photodynamic Therapy Using Solid Lipid Nanoparticles with Aluminum Phthalocyanine Chloride as a Nanocarrier for Modulating Immunogenic Cell Death in Murine Melanoma In Vitro

**DOI:** 10.3390/pharmaceutics16070941

**Published:** 2024-07-14

**Authors:** Marina M. Simões, Karen L. R. Paiva, Isadora Florêncio de Souza, Victor Carlos Mello, Ingrid Gracielle Martins da Silva, Paulo Eduardo Narcizo Souza, Luis Alexandre Muehlmann, Sônia Nair Báo

**Affiliations:** 1 Laboratory of Microscopy and Microanalysis, Department of Cell Biology, Institute of Biological Sciences, University of Brasília, Brasilia 70910-900, DF, Brazil; marinamesquita3007@gmail.com (M.M.S.); karendepaiva@gmail.com (K.L.R.P.); isadoraflorenciofs@gmail.com (I.F.d.S.); victor.silva@unb.br (V.C.M.); gracilias@gmail.com (I.G.M.d.S.); 2 Optical Spectroscopy Laboratory, Institute of Physics, University of Brasilia, Brasilia 70910-900, DF, Brazil; psouza@unb.br; 3 Laboratory of Nanoscience and Immunology, Faculty of Ceilandia, University of Brasilia, Brasilia 70910-900, DF, Brazil; luisalex@unb.br

**Keywords:** melanoma, B16-F10, photodynamic therapy, nanocarriers, immunogenic cell death, dendritic cells

## Abstract

Photodynamic therapy (PDT) uses a photosensitizer to generate reactive oxygen species (ROS) that kill target cells. In cancer treatments, PDT can potentially induce immunogenic cell death (ICD), which is characterized by a well-controlled exposure of damage-associated molecular patterns (DAMPs) that activate dendritic cells (DCs) and consequently modulate the immune response in the tumor microenvironment. However, PDT still has limitations, such as the activity of photosensitizers in aqueous media and poor bioavailability. Therefore, a new photosensitizer system, SLN-AlPc, has been developed to improve the therapeutic efficacy of PDT. In vitro experiments showed that the light-excited nanocarrier increased ROS production in murine melanoma B16-F10 cells and modulated the profile of DCs. PDT induced cell death accompanied by the exposure of DAMPs and the formation of autophagosomes. In addition, the DCs exposed to PDT-treated B16-F10 cells exhibited morphological changes, increased expression of MHCII, CD86, CD80, and production of IL-12 and IFN-γ, suggesting immune activation towards an antitumor profile. These results indicate that the SLNs-AlPc protocol has the potential to improve PDT efficacy by inducing ICD and activating DCs.

## 1. Introduction

Melanoma is a cancer that originates from melanocytes, the cells responsible for the production of the pigment melanin [1,2]. This type of skin cancer causes high mortality, and its incidence has been increasing in recent decades [3,4]. Current clinical treatment of melanoma includes surgery, radiotherapy, chemotherapy and, more recently, immunotherapy. However, conventional treatments still have limitations and disadvantages, such as severe side effects and high recurrence rates. There is therefore an increasing need for new strategies to improve the effectiveness of available therapies or to search for new therapeutic approaches against cancer. Among the alternative methods, photodynamic therapy (PDT) stands out [5,6,7].

PDT is an alternative non-invasive, local treatment for cancer. It is based on the production of oxidative species by a photoactivated dye, the photosensitizer, which is a molecule capable of converting specific light energy into chemical potential. Cytotoxic reactants, primarily reactive oxygen and nitrogen species, induce cell death in cancer cells and vascular blockage with subsequent tumor ischemia, and they can enhance the immune response to tumor antigens [8,9,10]. The optical properties of the skin, heavily influenced by melanin, are essential considerations when selecting photosensitizers (PSs) for PDT for melanoma. Melanin’s light-absorbing properties and antioxidant capacities can significantly affect the treatment response. Melanin exhibits higher absorption at shorter wavelengths, with a UV–Vis absorption spectrum showing a gradual increase in absorbance from 750 to 600 nm, a moderate increase from 600 to 450 nm, and a sharp increase from 450 nm to a broad peak around 335 nm [11,12,13]. Recognizing this issue, a good solution to overcome this disadvantage is the use of aluminum phthalocyanine chloride (AlPc), which absorbs light between 660 and 770 nm, away from the melanin absorbance peak [14]. AlPc offers additional advantages such as its high molar absorption coefficient, fluorescence emission, and ^1^O_2_ generation. Moreover, the disadvantages that this compound previously had, such as low water solubility and low bioavailability, are being overcome through the development of new photosensitizers using nanotechnology, which can enhance PDT [15].

Regarding the immune branch of PDT-related effects, despite several studies describing it, its mechanisms seem to involve both activation of immune effector cells and a reduced immunosuppressive microenvironment [14,16]. In this context, over the past few years it has become clear that the modality of cell death induced by PDT strongly influences the immune outcomes of this therapy [17]. The induction of immunogenic cell death (ICD) has been pointed out as one possible mechanism underlying the PDT-elicited immune response. Cancer cells undergoing ICD expose damage-associated molecular patterns (DAMPs), which attract and activate different immune cells, including antigen-presenting cells [18,19,20]. Among antigen-presenting cells, dendritic cells (DCs) play a crucial role in the immune system due to their ability to connect innate and adaptive immune responses. DCs have the unique capacity to transport tumor antigens to draining lymph nodes to initiate T-cell activation. Moreover, tumor-resident dendritic cells can also influence the regulation of T-cell responses in tumors during therapy [21,22,23]. Therefore, it is evident that interactions among different cell populations within tumors play a crucial role in disease development, and assessing how immunogenic cell death induced by photodynamic therapy can interfere with these interactions is essential for enhancing its efficiency.

The activation of the immune system due to the modulation of the tumor microenvironment through photodynamic therapy is related to parameters such as light dose, photosensitizer concentration, and the interval between administration of the photosensitizer and irradiation of the target tissue. Therefore, to enhance the potential of PDT, the use of nanoparticles has become a strategy to overcome limitations such as low photosensitizer bioavailability and poor photoactivity in aqueous media. The use of nanocarriers can promote greater therapy efficacy and increased immune system activation [14,24]. Therefore, this study aimed to evaluate the potential of PDT mediated by a lipid-based nanocarrier containing the photosensitizer aluminum phthalocyanine chloride (SLNs-AlPc) to increase the immunogenicity of murine melanoma B16F10 cells, thus activating dendritic cells.

## 2. Materials and Methods

### 2.1. Preparation of Solid Lipid Nanoparticles (SLNs)

The nanoparticles used in this study were prepared according to the process described by Mello et al. (2022) [25]. Solid lipid nanoparticles were prepared by a low-energy method. Murumuru butter (SISGEN:A1563A6, Amazon Oil, Ananindeua, Brazil) was selected as the solid lipid, and the selected surfactant was Brij™ O10 (Sigma-Aldrich, St. Louis, MO, USA), in a ratio of 2:1, corresponding to 5% (*w*/*v*) of the formulation, and the final concentration of the photosensitizer aluminum phthalocyanine chloride (AlPc) was 20 µM. The samples were developed by heating the organic phase—composed of butter, surfactant, and AlPc—and water at 75 °C, separately. After complete melting of the butters under magnetic agitation of 500 rpm, the aqueous phase was transferred under the organic, and agitation was maintained constant for 5 min. After this period, the system was subjected to magnetic agitation at 500 rpm, without heating, for 5 min until room temperature and formation of the SLNs were reached. They were analyzed in two different groups, solid lipid nanoparticles with aluminum phthalocyanine chloride (SLNs-AlPc) and solid lipid nanoparticles without the photosensitizer (SLNs).

### 2.2. Cell Culture

Murine melanoma B16-F10 cells were cultured in DMEM medium supplemented with 1% antibiotic solution (100 units/mL of penicillin and 100 μg/mL of streptomycin) and 10% fetal bovine serum (FBS). The cells were maintained in a humidified incubator at 37 °C with 5% CO_2_.

### 2.3. Cell Viability Assay

For the viability assay, B16-F10 cells were treated with two nanoparticles, SLNs-AlPc and SLNs, developed at the Nanotechnology Laboratory of the University of Brasília, as well as with the drug mitoxantrone (MTX), as it induces immunogenic cell death. In accordance with the literature, the cells were incubated with MTX diluted in DMEM medium for 30 min at 37 °C, then replaced with the same medium without MTX [14]. Cell viability was determined by the standard MTT assay following the manufacturer’s recommendations. A total of 3 × 10^3^ B16-F10 cells per well were seeded in 96-well plates. After adherence, the cells were treated with different concentrations of the nanocarriers (0.39, 0.78, 1.56, 3.12, 6.25, 12.5, 25, and 50 nM), or mitoxantrone (0.08, 0.15, 0.31, 0.62, 1.25, 2.5, 5 and 10 µM), or maintained only with culture medium. Subsequently, the cells were treated with the nanocarriers for 15 min. Then, the culture medium was replaced with complete growth medium. They were either kept in the dark or irradiated for 10 min with LED light (660 nm at 25.9 J/cm^2^ energy density). After a 24-h treatment period, the wells were incubated for 4 h in the dark at 37 °C with 150 µL of MTT solution (0.5 mg/mL in culture medium). Then, this solution was removed, and 200 µL of DMSO was added to each well to dissolve the formazan crystals. The plates were read on a Spectramax M5 spectrophotometer (Molecular Devices, LLC—San Jose, CA, USA).

### 2.4. Photodynamic Therapy Protocol

**Figure 1:**
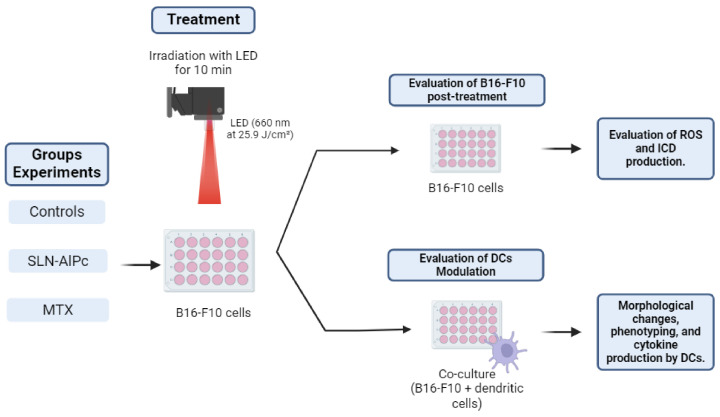
Illustrative scheme of the protocol used for photodynamic therapy in the experiments. The B16-F10 cells were treated with solid lipid nanoparticles with aluminum phthalocyanine chloride (SLNs-AlPc) or mitoxantrone (MTX). Reactive oxygen species (ROS), immunogenic cell death (ICD), dendritic cells (DCs).

Photodynamic therapy involves the use of a light-emitting diode (LED) array system to irradiate cells along with the SLNs-AlPc nanocarrier. For PDT treatment, cells were exposed to the nanocarrier for 15 min (IC50 = 1.7 nM). Subsequently, the cells were either kept in the dark or irradiated for 10 min with LED light (660 nm at 25.9 J/cm^2^ energy density). Positive controls for immunogenic cell death (ICD) consisted of B16-F10 cells treated with mitoxantrone (IC50 = 0.6 µM) for 24 h, as illustrated in Figure 1.

### 2.5. Quantification of ROS

The quantification of ROS production post-treatment was conducted using a Cellular ROS Assay Kit (Orange, Abcam, Cambridge, UK) on a FACSCalibur flow cytometer (BD, Franklin Lakes, NJ, USA). For this, 5 × 10^5^ cells were plated in 6-well plates and subsequently treated with PDT (SLNs-AlPc at 1.7 nM), MTX (0.6 µM), H_2_O_2_ (26 mM) as a positive control, or N-acetylcysteine (NAC) (10 mM) as a negative control; all treatments were diluted in DMEM medium. After a treatment period of 1 h, the cells were incubated with ROS Orange (Ex/Em = 540/570 nm) for 1 h at 37 °C. Then, the cell lines underwent the processes of detachment and washing in PBS 1X. After these steps, they were analyzed by flow cytometry, FACSCalibur (BD, USA).

### 2.6. Morphological Analysis by Transmission Electron Microscopy

The cells were plated in 6-well plates at a density of 1 × 10^6^ cells per well. After adherence, the cells were treated with MTX (0.6 µM) or irradiated with PDT (SLNs-AlPc at a concentration of 1.7 nM); both treatments were diluted in DMEM. Subsequently, the cells were detached from the wells with trypsin, washed with PBS, and fixed overnight in Karnovsky fixative (4% paraformaldehyde and 2% glutaraldehyde in 0.1 M sodium cacodylate buffer, pH 7.2). They were then washed in the same buffer, post-fixed for 30 min in 1% osmium tetroxide and 0.8% potassium ferricyanide in 0.1 M sodium cacodylate buffer, pH 7.2, dehydrated gradually in acetone (30–100%), and embedded in Spurr resin. Ultra-thin sections obtained with an ultramicrotome (Leica Microsystems, Wetzlar, Germany) were contrasted with uranyl acetate and analyzed with a Jeol 1011 Transmission Electron Microscope (Jeol, Peabody, MA, USA) at an acceleration voltage of 80 kV.

### 2.7. Assays for Immunogenic Cell Death

#### 2.7.1. Confocal Microscopy

B16-F10 cells were seeded over round coverslips in 24-well plates at a concentration of 1 × 10^5^ cells/well. After adherence, the cells were treated with either PDT (SLNs-AlPc at a concentration of 1.7 nM) or mitoxantrone at a concentration of 0.6 µM/mL, both diluted in DMEM. After 24 h, the cells were washed with PBS and fixed with 3.7% formaldehyde for 30 min. Blocking solution (1% skim milk, 2.5% bovine serum albumin (BSA), 8% fetal bovine serum (FBS) in PBS) was added and left for 20 min, followed by incubation overnight at 4 °C, with primary mouse anti-HMGB1 or anti-calreticulin antibody diluted in the blocking solution. Wells were washed with PBS, and the secondary antibody Alexa-488 or Alexa-647 anti-mouse (5 µg/mL) diluted in PBS, respectively, was added for 1 h at 37 °C, protected from light. Wells were then washed with PBS and incubated for 5 min with DAPI (300 nM) for cellular DNA staining. Wells were washed with PBS, and coverslips were mounted with ProLong Gold Antifade and analyzed using a TCS SP5 Laser Scanning Confocal Microscope (Leica Microsystems, Germany).

#### 2.7.2. Transmission Electron Microscopy

To observe the localization of calreticulin after treatment with PDT, 2 × 10^6^ cells were seeded per well in 6-well plates and treated with SLNs-AlPc at a concentration equivalent to 1.7 nM AlPc and with MTX at a concentration of 0.6 µM, both diluted in DMEM, for 24 h, while the control received no treatment but only medium replacement. The cells were fixed (4% paraformaldehyde, 0.5% glutaraldehyde, and 0.2% picric acid in 0.1 M sodium cacodylate buffer, pH 7.2) at 4 °C for 2 h. The material was washed with sodium cacodylate buffer and immersed in blocking solution (50 mM ammonium chloride) twice for 30 min each. Then, 2% uranyl acetate in 15% acetone was added to the samples in the dark for 2 h. Subsequently, the cells were dehydrated in acetone (30–90%) at 4 °C and embedded in LR Gold at −20 °C under ultraviolet light. Ultra-thin sections of 50 nm to 70 nm were obtained with diamond knives on an ultramicrotome (Leica Microsystems, Germany). The sections were mounted on nickel grids. The samples were then immersed in blocking solution (1% BSA and 0.01% Tween 20) for 1 h at room temperature. Immunostaining was performed with primary anti-calreticulin antibody in a ratio of 1:10 in deionized water for 1 h, and secondary antibody conjugated with 10 nm gold in a ratio of 1:20 in deionized water for 1 h. The samples were examined with a Jeol 1011 Transmission Electron Microscope (Jeol, Peabody, MA, USA) at an acceleration voltage of 80 kV.

### 2.8. Obtaining Precursor Cells and Culture of Murine Dendritic Cells

Dendritic cells (DCs) were obtained from the bone marrow of C57BL/6 mice. Animal handling was conducted following the guidelines of the Ethics Committee (046/19). For the in vitro study, bone marrow cells isolated from femurs and tibias were cultured for 7 days at a density of 2 × 10^6^ per 10 cm dish (10 mL) in a medium henceforth referred to as “complete differentiation medium”. This culture medium consisted of RPMI supplemented with 10% FBS (PAA Laboratories, Pasching, Austria), 1% *v*/*v* antibiotic (penicillin 10,000 units/mL, streptomycin 10,000 μg/mL Gibco, Grand Island, NE, USA), GM-CSF (20 ng/mL—Sigma-Aldrich, St. Louis, MO, USA), β-mercaptoethanol (50 μM—Sigma-Aldrich, St. Louis, MO, USA), and IL-4 (20 ng/mL—Sigma-Aldrich, St. Louis, MO, USA). The cells were maintained in sterile Petri dishes in a humidified incubator with 5% CO_2_ and 95% air at 37 °C. On day 3, an additional 10 mL of fresh complete differentiation medium was added. The cells were further differentiated for 4 more days. After this period, the floating cells were separately examined for their CD11c surface marker expression by flow cytometry using a FACSCalibur system (BD, Franklin Lakes, NJ, USA).

### 2.9. Morphological Analyses of the Co-Culture

#### 2.9.1. Light Microscopy

To assess morphological changes in dendritic cells in co-culture with the B16-F10 cell line, 5 × 10^4^ B16-F10 cells were seeded in 24-well plates. The protocol of the experiments followed the method described in Section 2.3, and each treatment was executed independently. After cell adhesion, they were treated with concentrations of PDT (SLNs-ALPc = 1.7 nM), MTX (0.6 µM), and LPS (1 µg/mL) as a positive control for dendritic cell activation. Following treatment, dendritic cells were added at a 1:1 ratio, and the cells were maintained in a humidified incubator at 37 °C coupled with an Evos Microscope (Thermo Fisher Scientific, Waltham, MA, USA), which was used for analysis through time-lapse imaging over a 24-h period, at 10× magnification.

#### 2.9.2. Scanning Electron Microscopy

In order to analyze the morphological differences present on the cell surface as a result of photodynamic therapy, tumor cells and dendritic cells were examined using scanning electron microscopy (SEM). Initially, 5 × 10^4^ tumor cells were plated on 18 × 18 mm coverslips placed in 6-well plates. The following day, the cells were treated with PDT (SLNs-AlPc = 1.7 nM) and then co-cultured with dendritic cells. After 15, 30, 45 min, and 24 h of co-culture, the culture medium and treatment were discarded, and the cells underwent a washing process with PBS 1X 3 times. They were then fixed in 2% paraformaldehyde, 2% glutaraldehyde in 0.1 M sodium cacodylate buffer, pH 7.2, overnight at 4 °C. Following fixation, the cell lines were washed once with 0.1 M sodium cacodylate buffer and post-fixed for 30 min with 1% osmium tetroxide diluted in 0.1 M sodium cacodylate buffer, pH 7.2, with deionized water. The cells underwent serial dehydration with increasing concentrations of acetone (30–100%), were dried using a critical point dryer CPD 030 (BALZERS, Hudson, NH, USA), and were metalized with SCD 500 (LEICA, Germany) before being analyzed under a JSM-7001F Scanning Electron Microscope (JEOL, Akishima, Japan).

### 2.10. Dendritic Cell Maturation

For the study of dendritic cell maturation, cells were co-cultured with B16-F10 cells. The protocol of the experiments was carried out according to the method described in Section 2.3, and each treatment was executed independently. After cell adhesion, they were treated with concentrations of PDT (SLNs-AlPc = 1.7 nM), MTX (0.6 µM), and LPS (1 µg/mL) as a positive control for pro-inflammatory profile, for 24 h at a 1:1 ratio at 37 °C (total cells = 1 × 10^6^). After this co-culture period, cells were detached using a cell scraper and washed with PBS, and a blocking solution (2.5% BSA) was added for 15 min. Then, cells were labeled with anti-CD11c, CD86, CD80, and MHCII antibodies for 1 h on ice and analyzed by flow cytometry, using the FACSCalibur system (BD, USA). The anti-CD11c marker was used to gate the dendritic cell-enriched population, and the other markers were analyzed within this selected subpopulation.

### 2.11. Analysis of Cytokine Production by Dendritic Cells after Co-Culture with Tumor Cells Treated with PDT

B16-F10 cells were seeded in 6-well plates at 5 × 10^5^ cells/well, and after 24 h, they were treated with MTX (0.6 µM) or treated with PDT (SLNs-AlPc = 1.7 nM) and were kept in the dark. After treatment for 24 h, dendritic cells were added to the wells in a 1:1 ratio. As a positive control for the activation of a pro-inflammatory profile of dendritic cells, cells were treated with LPS (1 µg/mL) for 24 h. Then, at the end of the specified co-culture time, supernatants were collected to map cytokine production stimulated by the treatments, using an ELISA Kit. The cytokines IL-12, IL-10, TNF-α, and IFN-γ were analyzed with commercial kits from BD Biosciences according to the manufacturer’s protocols. Absorbance values were generated from readings on a spectrophotometer (Varioskan—ThermoFisher, Waltham, MA, USA) at a wavelength of 450 nm. The determination of cytokine concentrations was performed using specific standard curves for each cytokine, presented as absolute values in picograms per milliliter (pg/mL).

### 2.12. Statistical Analysis

The data from the analyses were subjected to analysis of variance (ANOVA). Statistical analyses were conducted using GraphPad Prism 9 software, and the results are presented as mean ± SEM. Significance level (α) in this work was set at *p* < 0.05.

## 3. Results

### 3.1. Cell Viability Assay

**Figure 2:**
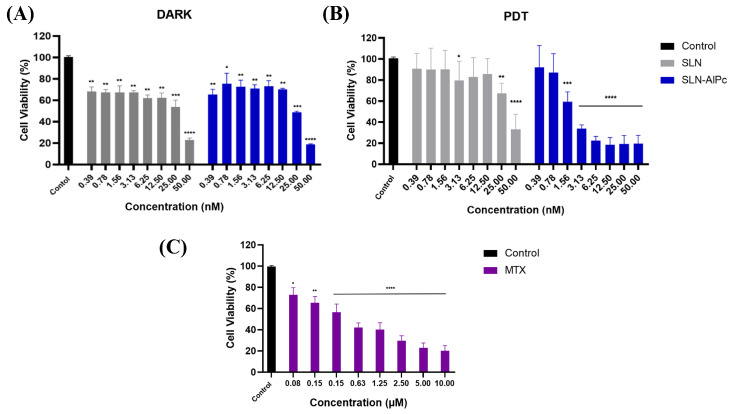
Analysis of B16–F10 cell viability by MTT assay with treatments after 24h. (**A**) Viability graph of B16-F10 lineage after SLN and SLN-AlPc treatments following 24 h of treatment. (**B**) Viability graph of B16-F10 lineage after treatment with photodynamic therapy, using SLNs and SLNs-AlPc after 24 h of treatment. (**C**) Analysis of B16-F10 cell viability by MTT assay after treatment with mitoxantrone after 24 h. Bars represent cell viability as a percentage after treatments at the indicated concentrations. Data represent the mean ± SEM of three independent experiments in triplicate. * *p* < 0.05, ** *p* < 0.01, *** *p* < 0.001, and **** *p* < 0.0001 compared with untreated control.

Through the MTT assay on B16-F10 cells, it was possible to evaluate the cytotoxicity of the SLNs-AlPc and MTX. After 24 h of treatment, Figure 2 shows that SLNs demonstrated low cytotoxicity in vitro, while SLNs-AlPc, when kept in the dark, exhibited a pattern similar to that of the nanocarrier without the photosensitizer. On the one hand, as expected, irradiation of SLNs did not increase their cytotoxicity (Figure 2B). On the other hand, irradiation of SLNs-AlPc significantly increased their cytotoxicity. This suggests that the nanostructure containing the photosensitizer resulted in a significant decrease in cell viability only when irradiated, indicating that the photosensitizer became more toxic only when excited by LED. The assay allowed for the calculation of the concentration needed to reduce the cell population by 50% (IC_50_), and the values obtained for SLNs-AlPc were 1.7 nM and for MTX were 0.6 µM (Figure 2C). These concentrations were used in subsequent treatments to evaluate the difference between the treatments.

### 3.2. Quantification of ROS

**Figure 3:**
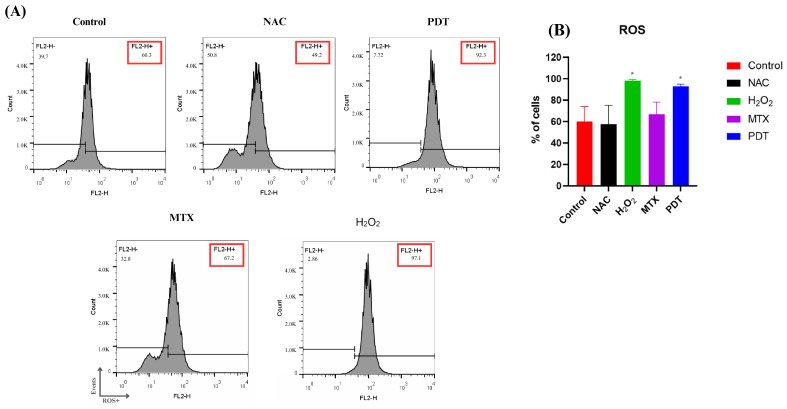
Analysis of ROS production in B16-F10 cells after treatments. (**A**) Representative histogram of reactive oxygen species production by B16-F10 cells pre- and post-treatments. (**B**) Quantification of the percentage of cells in each treatment group after 2 h. Cells were treated with NAC (negative control), H_2_O_2_ (positive control), photodynamic therapy (PDT), and mitoxantrone (MTX). The results presented are from three independent experiments, with the average percentage of cells in each treatment group ± SEM. * *p* < 0.05 compared with control.

ROS production was measured by flow cytometry two hours after application of the treatment. Hydrogen peroxide (H_2_O_2_) was used as a positive control and N-acetyl-L-cysteine (NAC) as a negative control (Figure 3).

In Figure 3B, it is possible to observe that the production of ROS reached a higher peak in the group treated with H_2_O_2_, followed by the group treated with PDT. 

### 3.3. Evaluation of Immunogenic Cell Death Mediators and Morphological Changes by Transmission Electron Microscopy

To better understand how photodynamic therapy might induce changes in the tumor microenvironment, two proteins, calreticulin and HMGB1, were selected to evaluate the potential of the therapy to induce immunogenic cell death.

**Figure 4:**
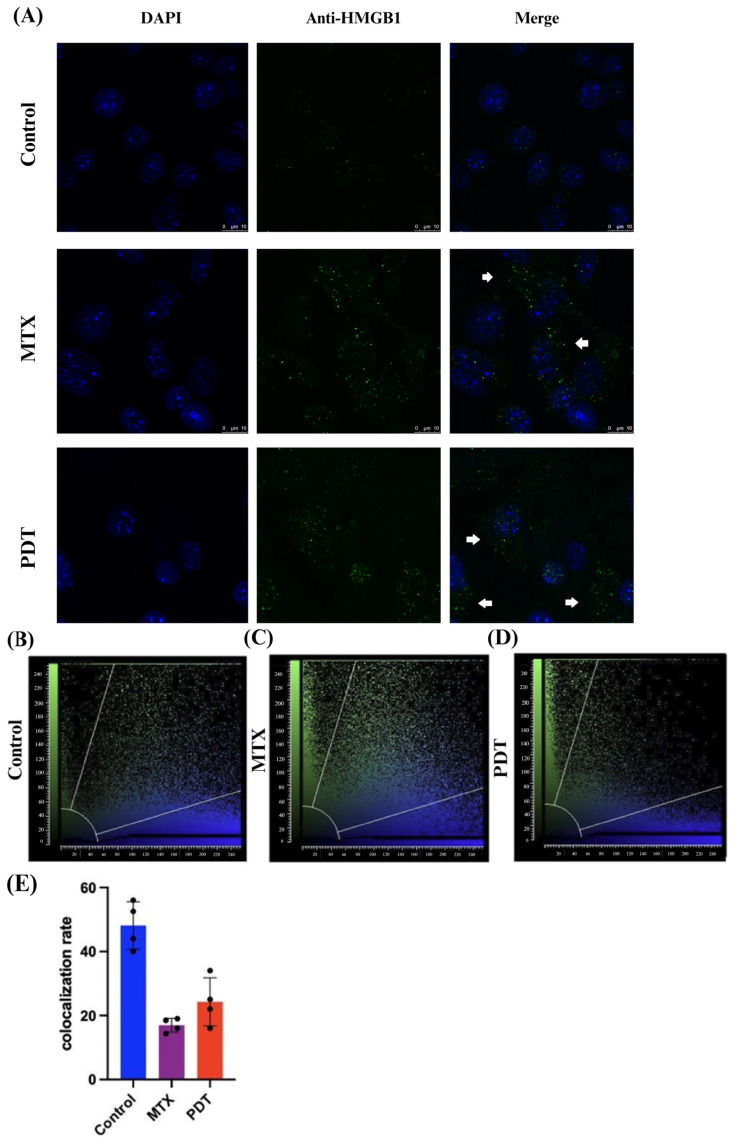
Immunostaining of HMGB1 and quantitative fluorescence analysis in B16-F10 cells after 24 h of treatment. (**A**) DAPI staining the nucleus in blue, green marking the HMGB1 protein, and the overlay of both labels. Arrows indicate the extravasation of HMGB1 from the nucleus and its presence in the cellular cytoplasm. The 3D reconstruction videos can be found in the Supplementary Material (Video S1, Video S2, and Video S3). (**B**–**D**) Co-localization plots of the 3D reconstructions, where the blue color represents voxels positive for the nucleus (DAPI), and the green color represents voxels positive for HMGB1. Points present in the intermediate region of the plot represent co-localized voxels. The quantification of co-localization is depicted in (**E**).

**Figure 5:**
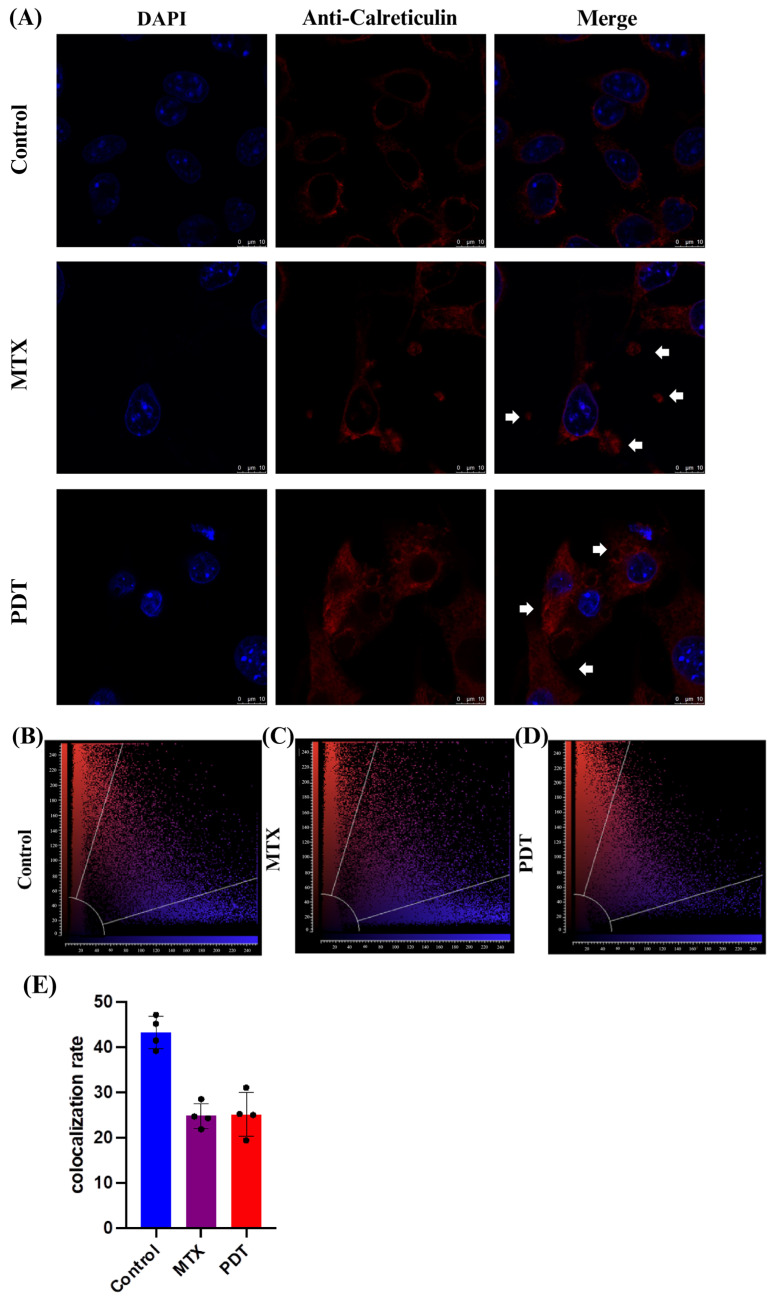
Immunostaining of calreticulin and quantitative fluorescence analysis in B16-F10 cells after 24 h of treatment. (**A**) DAPI staining the nucleus in blue, red marking the calreticulin protein, and the overlay of both labels. Arrows indicate the increased presence of calreticulin near the cell membrane and the formation of vesicles containing this protein. The 3D reconstruction videos can be found in the Supplementary Material (Video S4, Video S5, and Video S6). (**B**–**D**) Co-localization plots of the 3D reconstructions, where the blue color represents voxels positive for the nucleus (DAPI), and the red color represents voxels positive for calreticulin. Points present in the intermediate region of the plot represent co-localized voxels. The quantification of co-localization is depicted in (**E**).

Through the confocal microscopy technique, it was observed that after 24 h of treatment there was an extravasation of the HMGB1 protein, stained in green, from the cell nucleus to the cytoplasm in the groups treated with PDT and MTX (Figure 4). Images obtained from cells stained with anti-calreticulin showed a change in the protein’s staining pattern. In the group treated with PDT, changes in the cell’s structural arrangement were seen, and the group treated with MTX showed vesicle formation with calreticulin (Figure 5A). Furthermore, in the control group, the protein stained in red was more perinuclear, presenting a higher colocalization rate (Figure 5E). However, in the treated groups, there was a noticeable increase in fluorescence intensity closer to the cell membrane.

**Figure 6:**
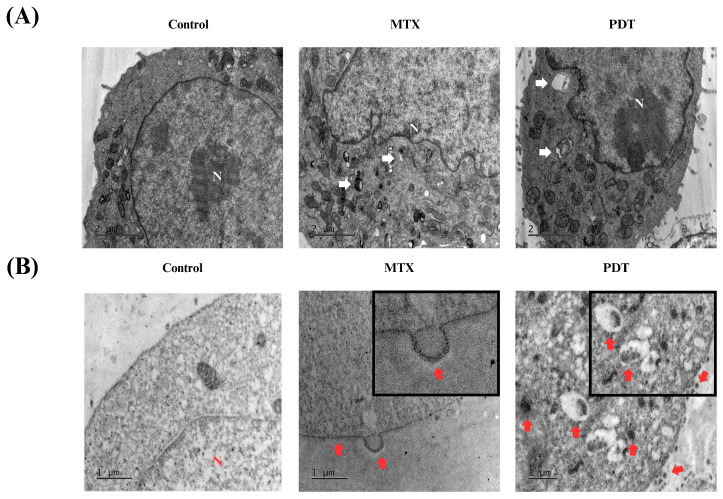
Analysis of morphological changes and immunostaining by transmission electron microscopy of B16-F10 cells after 24 h of treatment. Images obtained by transmission electron microscopy after 24 h treatment of B16-F10 cells with photodynamic therapy (PDT) and mitoxantrone (MTX). (**A**) Observation of autophagosomes after treatment, indicated by white arrows. (**B**) Immunostaining for the protein calreticulin, red arrows indicate the translocation of this protein into the cell membrane of B16-F10 cells after treatment. N indicates the cell nucleus.

In addition to these analyses, transmission electron microscopy assays were also performed to further investigate the cellular changes induced by the treatments and the pattern of calreticulin localization. In Figure 6A, the formation of autophagosomes is visible in both the PDT and MTX treated groups. In the immunostaining photomicrographs (Figure 6B), it was observed that in the group treated with photodynamic therapy, there was an accumulation of calreticulin in cytoplasmic regions that later translocated to expose this protein on the cell membrane. In cells treated with MTX, an accumulation of calreticulin on the cell membrane and the formation of vesicles containing this protein were also observed. These vesicles could have a signaling function for other cells in the tumor microenvironment.

### 3.4. Acquisition of Precursor Cells and Culture of Enriched Dendritic Cell Population

The enriched population of dendritic cells (DCs) was obtained from the bone marrow of C57BL/6 mice. For the in vitro study, bone marrow cells isolated from femurs and tibias were cultured for 7 days as described in the methodology. After this period, the floating cells were analyzed separately via flow cytometry for their expression of the surface marker CD11c.

**Figure 7:**
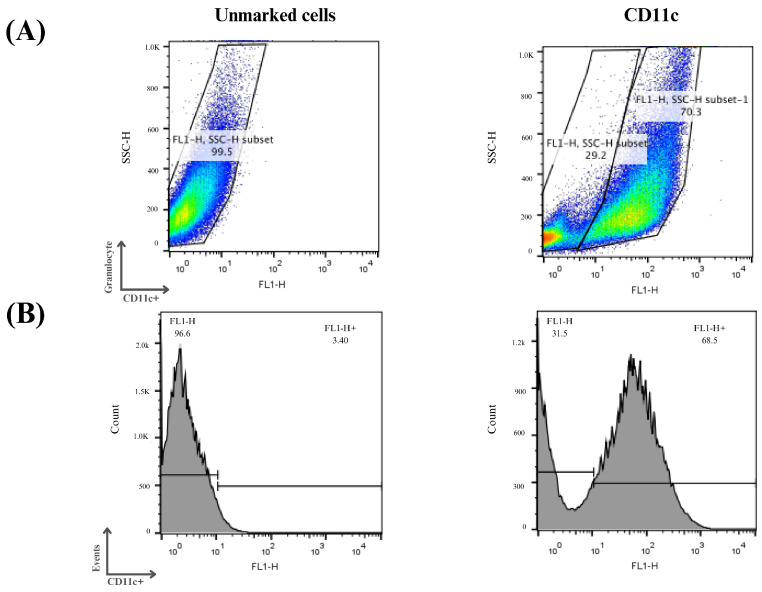
Flow cytometry analysis of monocyte differentiation into dendritic cells. Mouse bone marrow-derived cells were subjected to a cellular differentiation process to obtain immature dendritic cells, which were used for this study. (**A**) Cell distribution according to the labeling profile after the differentiation period. (**B**) Histograms of the cells obtained after the differentiation period. Cells that were positive for the CD11c marker were considered immature dendritic cells.

Three independent assays were performed, and it can be seen in Figure 7 that on average 70% of the cells examined showed an increase in CD11c expression, indicating the process of cellular differentiation of bone marrow precursor cells into immature dendritic cells. The differentiation and cell culture results obtained were considered satisfactory and the same protocol was used for the other analysis in this study.

### 3.5. Morphological Analysis of Co-Culture

After obtaining the enriched population of dendritic cells, we started studies with these cells to understand how photodynamic therapy could affect their activation. The first tests were performed to analyze the morphology of these cells in co-culture with the B16-F10 cell line after the treatments.

**Figure 8:**
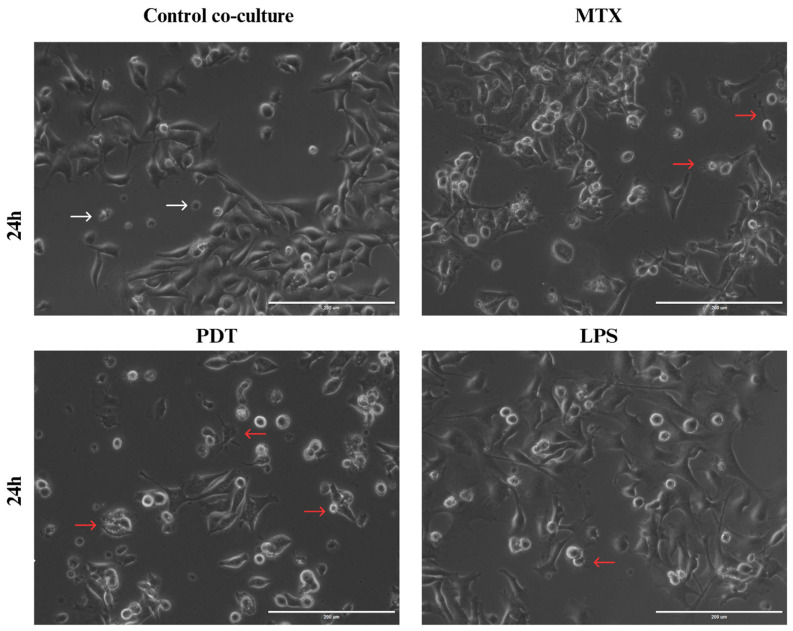
Light microscopy of B16-F10 cells after 24 h of treatments co-cultured with DCs. Observation of dendritic cell (DC) changes in co-culture with B16-F10 cells treated with photodynamic therapy (PDT), mitoxantrone (MTX), and lipopolysaccharide (LPS), 24 h after the start of co-culture. White arrows indicate dendritic cells without alterations that maintain their spherical shape, while red arrows indicate dendritic cells with altered morphology that show elongation of dendrites and interaction with B16-F10 cells.

Through light microscopy, it was possible to observe the initial signs of morphological changes in the enriched population of dendritic cells in each treatment group over the analysis times (Figure S1 in the Supplementary Information). In the images acquired at the 24 h time point (Figure 8), an increase in cellular dendrites and a decrease in the spheroid shape of dendritic cells were observed in the groups treated with PDT and MTX. In addition, dendritic cells with altered morphology were found near tumor cells undergoing cell death following these treatments. Compared with the cells cultured with the control and the dendritic cells maintained in culture alone, the morphological pattern observed included a higher presence of spheroidal cells with reduced or virtually absent dendrites.

**Figure 9:**
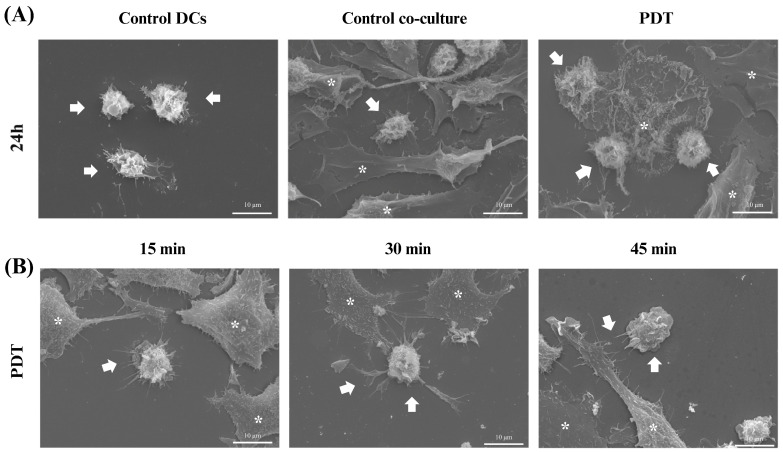
Scanning electron microscopy of dendritic cells co-cultured with B16-F10 cells after treatments. Observation of changes in dendritic cells (DCs) co-cultured with B16-F10 cells treated with photodynamic therapy (PDT). (**A**) Images of DCs alone or in co-culture after 24 h of PDT treatment. (**B**) Images of cellular co-culture after PDT treatment at 15, 30, and 45 min. The arrows indicate dendritic cells and the asterisks represent the B16-F10 cells.

Scanning electron microscopy (SEM) was used to gain further insight into the morphological characteristics of the cells after 24 h of co-culture. In the microscopy images acquired after treatment with photodynamic therapy, it was noticeable that both the size of the dendritic cells and their protrusions had increased and there was a greater interaction with B16-F10 cells undergoing cell death (Figure 9A), which was consistent with the results of the light microscopy. In addition, an analysis of these changes was also performed at three initial time points after PDT treatment: 15 min, 30 min, and 45 min. Within these intervals, it was already possible to see that the treatment induced morphological changes such as enlargement of the cellular cytoplasm, elongated structures, and increased projections on the dendritic cells (Figure 9B).

### 3.6. Activation of the Enriched Dendritic Cell Population

**Figure 10:**
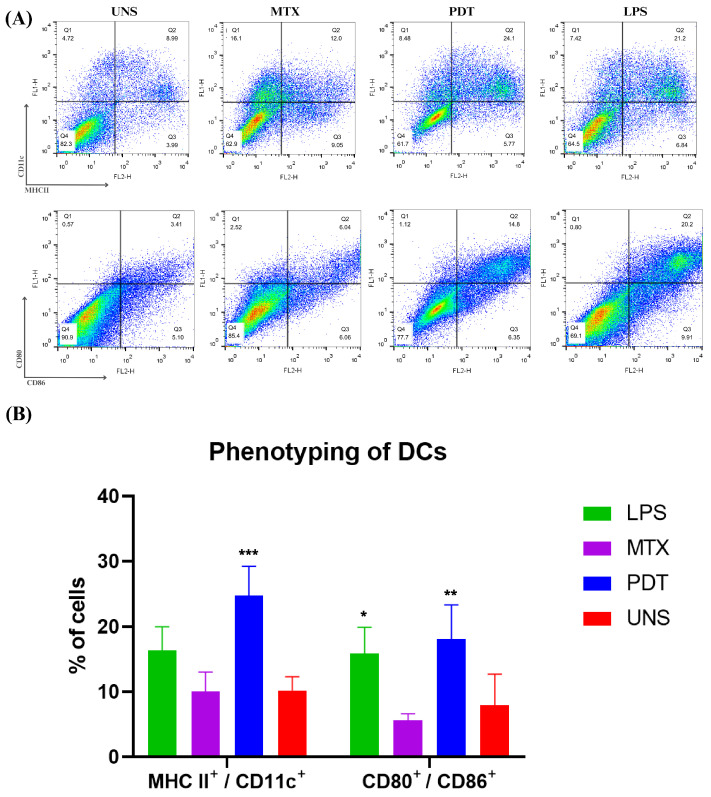
Dendritic cell phenotyping analysis after 24 h of cell co-culture. (**A**) Dot plot of dendritic cells co-cultured with B16-F10 cells in each treatment group after 24 h. The groups included cells treated with lipopolysaccharide (LPS), mitoxantrone (MTX), photodynamic therapy (PDT), and unstimulated cells (UNS). The markers used were CD11c, MHCII, CD80, and CD86. (**B**) Quantification and graphical representation of the percentage of dendritic cells co-cultured with B16-F10 cells in each treatment group after 24 h. The presented results are from three independent experiments, with the average percentage of cells in each treatment ± SEM. * *p* < 0.05, ** *p* < 0.01, and *** *p* < 0.001 compared with the control.

The maturation of dendritic cells is a crucial phase in their life cycle and involves several morphological and functional changes. After the morphological analyses by microscopy, an experiment was performed to investigate the expression and regulation of cell membrane markers related to the maturation and activation profile of these cells of the immune system. Thus, the co-cultured cells were phenotyped via flow cytometry and the molecules analyzed were CD11c, MHCII, CD80, and CD86 (Figure 10A). The results show that PDT increased the expression of these molecules compared with the control and the groups treated with MTX. Cells treated with PDT showed a significant increase, with the percentage of double-labelled cells being even higher than in the LPS group, which was used as a positive control (Figure 10B).

### 3.7. Cytokine Production by Dendritic Cells after Co-Culture with Tumor Cells Treated with PDT

**Figure 11:**
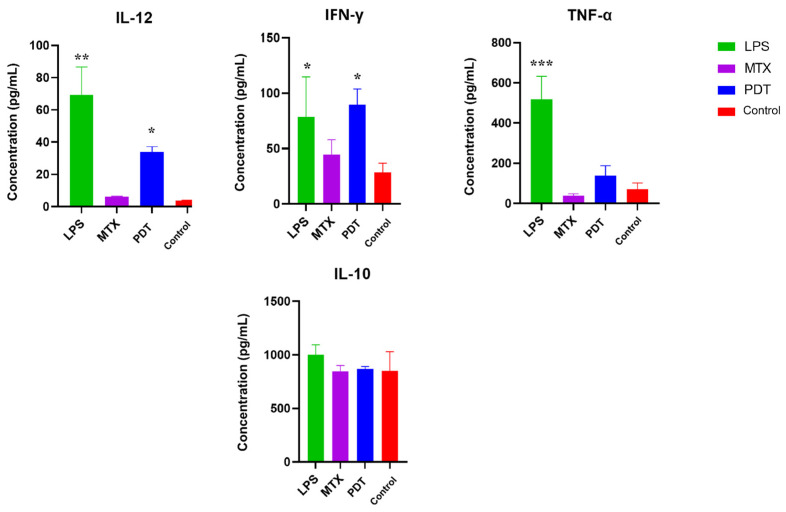
Analysis of cytokine production by dendritic cells and B16-F10 cells after 24 h of treatment. Quantification of cytokines after 24 h of treatment with photodynamic therapy (PDT), mitoxantrone (MTX), and lipopolysaccharide (LPS). The cytokines analyzed were IL-12, TNF-α, IL-10, and IFN-γ. Data represent the mean ± SEM of three independent experiments performed in triplicate. * *p* < 0.05, ** *p* < 0.01, and *** *p* < 0.001 compared with untreated control.

Cytokine screening by ELISA was performed to better understand the pattern of molecules stimulated after photodynamic therapy in the co-culture of melanoma cells with dendritic cells. The cytokines mapped were IL-12, TNF-α, IL-10, and IFN-γ. Figure 11 shows an increase in the production of IL-12, TNF-α and IFN-γ, which are considered pro-inflammatory cytokines, in the groups treated with PDT compared with the control group. IL-12 and IFN-γ showed a significant increase compared with the control, and IFN-γ showed a concentration very close to that of LPS, which was used as a positive control for dendritic cell activation. As for the production of anti-inflammatory cytokines such as IL-10, the concentration pattern remained similar between the groups, meaning there were no significant differences between them.

## 4. Discussion

Previous results published by this group showed that SLNs-AlPc are an effective third-generation photosensitizer, able to maintain the activity of AlPc, a hydrophobic compound, in an aqueous environment [25]. Considering the characterization results of the nanostructure, the present study aimed to evaluate their potential to induce immunogenic cell death (ICD) and to modulate the activity of dendritic cells (DCs) against PDT-treated B16-F10 cells.

The cell viability assay showed that the tumor cell line was sensitive to photodynamic therapy (PDT). An important factor to highlight is that when the tumor cells were treated with the nanocarrier and kept in the dark, low cytotoxicity (~70% viable cells) was observed, suggesting that the photo-oxidative effect of the photosensitizer only occurred when stimulated by the specific wavelength of light. Furthermore, the fact that SLNs-AlPc exhibited a cytotoxic concentration of 50% at 1.7 nM enabled cell death at a lower concentration of photosensitizer compared with other studies [26].

PDT generates oxidative stress, which can lead to different types of cell death, such as apoptosis, necrosis, and cellular autophagy [27]. Thus, the ROS production was assessed by flow cytometry. As expected, it was found that the PDT treatment increased the production of ROS. In the PDT group, over 90% of the cells were positively labeled for ROS, demonstrating the great potential of the nanosystem developed to combat melanoma. An excess of these oxidative components damages organelles, suppresses cell proliferation, inhibits the cell cycle, and leads to the shutdown of the tumor vasculature [28].

Oxidative stress can cause different types of cell death depending on the protocol chosen for PDT treatment. In the previous study with SLNs-AlPc, Mello et al. (2022) [25] showed that the protocol used led to the induction of apoptotic cell death with an increase in caspase-3 expression and a decrease in Bcl-2 expression. The presence of activated apoptosis markers together with the exposure of DAMPs is a hallmark of ICD. In the experiments performed to evaluate the production of ICD mediators, it was observed by confocal fluorescence microscopy that PDT induced an increase in the exposure of DAMPs. The microscopic images obtained showed that the PDT treatment caused the translocation of the non-histone protein HMGB1 from the nucleus to the cytoplasm. This result was consistent with the result obtained with the drug mitoxantrone (MTX), which was used as a positive control. The therapy also resulted in an alteration in the localization of calreticulin (CRT). Compared with the control, an increase in the intensity of the fluorescent marker was observed in regions further away from the nucleus and closer to the cell membrane. Another interesting event was the formation of vesicles containing this protein in the groups treated with MTX. These results support the idea that PDT treatment can induce adaptive immune responses by triggering ICD [29]. The presence of CRT on the surface of the cell membrane is known as “eat-me” signaling, which, when accompanied by the release of HMGB1, can activate antigen-presenting cells such as DCs. These cells possess toll-like receptors that can interact with these DAMPs and release pro-inflammatory factors that induce DCs’ maturation [30].

On the microscopic images obtained via immunostaining under electron microscopy, an accumulation of calreticulin at specific sites of the cytoplasm and the direction of these clusters to the plasma membrane region was observed after PDT treatment. In the cells treated with MTX, an accumulation of calreticulin at the cell membrane and the formation of vesicles were also observed. Thus, the images obtained by transmission electron microscopy were consistent with the results of fluorescence microscopy and demonstrated the potential of the therapy to induce the release of DAMPs.

In the transmission electron microscopy images, the presence of autophagosomes was also observed in the PDT-treated cells. Autophagy is a cellular process involved in the maintenance of homeostasis and is activated under stress conditions to recycle degraded cellular components [31]. Its role in cancer treatment is controversial, as it can both induce the death of tumor cells and promote their survival. PDT generates stress through reactive oxygen species that damage organelles such as the endoplasmic reticulum and mitochondria, and autophagy is a natural cellular mechanism that attempts to reverse this condition [32]. However, high production of ROS and a high level of autophagy can favor the pro-death process, as the cellular stress is so high that the cell can no longer achieve homeostasis. Thus, the autophagic apparatus can also contribute to other cell death programs such as apoptosis or necroptosis, leading to a form of autophagy-mediated cell death (AMCD). The autophagic process may be correlated with a higher production of immunogenic cell death signals, as vesicles can be released with signals for the immune system, such as calreticulin. According to Pietro et. al. (2020) [33], early inhibition of autophagy can attenuate the exposure of CRT. Thus, the high presence of autophagosomes and ROS production generated by photo-oxidative stress after the application of PDT with SLNs-AlPc could regulate the autophagic process towards a pro-death profile, with the release of DAMPs, and play a fundamental role in increasing death mediators, probably leading to AMCD along with apoptosis [33,34].

Subsequently, assays were performed with an enriched population of dendritic cells obtained by differentiation of bone marrow precursor cells from C57BL/6 mice. The images obtained by light microscopy evidenced that DCs co-cultured with PDT-treated B16-F10 cells exhibited changes in both their behavior and morphology. Compared with the control group, the DCs from the PDT group showed an elongated shape with the presence of extensions. These images supported the results of the scanning electron microscopy, which allowed a more detailed visualization of the morphological changes. Immature dendritic cells (imDCs) exhibited more spherical features with few projections, while mature dendritic cells (mDCs) showed a star-shaped morphology with long (>10 µm) and slender cytoplasmic projections that were spiky or leaf-like (actin-rich protrusions) [35]. The reorganization of the cell structure of the mDCs visible in the microscopic images was directly related to the increased motility and phagocytic processes. Therefore, these features could indicate the recognition, phagocytosis, and presentation of tumor antigens by dendritic cells, aiming at enhanced activation of the innate immune system and the adaptive immune system and communication between them.

In addition to morphological changes, the expression of co-stimulatory molecules is extremely important for the assessment of DC phenotyping. The results obtained by flow cytometry showed that PDT stimulated an increase in the expression of these molecules on the cell membrane and the major histocompatibility complex. In the mature phenotype, dendritic cells exhibit increased expression of MHC II and molecules such as CD80 and CD86, which are present on the membrane for antigen presentation and T-cell activation [36,37,38]. The data found in this study are consistent with descriptions by other authors [39] who also observed this pattern of molecular expression in mature dendritic cells (mDCs). Thus, photodynamic therapy mediated by SLNs-AlPc was able to trigger the maturation of dendritic cells [40].

The cytokine profile of the cell co-culture is also an important parameter for gaining a better understanding of how PDT can influence DCs and consequently alter the tumor microenvironment [41]. The results obtained by ELISA from the co-culture supernatant showed an increase in the concentrations of IL-12, TNF-α, and IFN-γ in the groups treated with PDT compared with the control group, while the concentration of IL-10 remained similar in all groups. The significant increase in IL-12 levels from the co-culture supernatant may be related to the modulation of cytokine release following the interaction between cancer cells and DCs. The tumor microenvironment alters the secretion profile of DCs, which under normal conditions synthesize IL-12 to enhance the anti-tumor immune response and increase immune surveillance to combat tumor cells [42]. Indeed, the observed increase in IL-12 levels in the results suggests that DCs become activated by exposure to PDT-treated cells.

The pro-inflammatory mediators TNF-α and IFN-γ also play an important role in the communication and activation of adaptive immunity, especially by stimulating Th1 cells, which are important for the elimination of cancer cells. In addition, IFN-γ production is associated with IL-12 production and regulates NK cells, which act in the control of tumor metastasis [43,44]. The increase of these cytokines after PDT was also described by Zhang et. al. (2022) [45], suggesting that ICD induced by PDT may enhance the anti-tumor immune response [46]. The results of this study showed that the concentration of IL-10 did not change between groups and that the production of this cytokine was not sufficient to prevent the production of IL-12 and IFN-γ, which are criteria for evaluating the development of mCDs [47].

## 5. Conclusions

This study suggests that SLNs-AlPc induced ICD in the B16-F10 melanoma cell line, which thus became able to activate DC. This nanosystem could thus be used in immunotherapy protocols against melanoma. The developed nanocarrier was able to increase the production of reactive oxygen species and induce immunogenic cell death of the investigated tumor cells. Photodynamic therapy mediated by SLNs-AlPc proved to be important for the modulation and activation of dendritic cells and could therefore be a promising treatment, as it may lead to the activation of the immune system to recognize and fight tumor cells near the primary tumor or in distant micrometastases.

## Data Availability

Data is contained within the article or Supplementary Material.

## Supplementary Materials

The following supporting information can be downloaded at: https://www.mdpi.com/article/10.3390/pharmaceutics16070941/s1, Figure S1: Light Microscopy of B16-F10 cells after treatments co-cultured with DCs; Video S1: The 3D reconstruction video of control HMGB1; Video S2: The 3D reconstruction video of the effect of PDT on HMGB1; Video S3: The 3D reconstruction video of the effect of MTX of HMGB1; Video S4: The 3D reconstruction video of control calreticulin; Video S5: The 3D reconstruction video of the effect of PDT on calreticulin; Video S6: The 3D reconstruction video of the effect of MTX on calreticulin.
