# Supplementary material for: The Potential of Photodynamic Therapy Using Solid Lipid Nanoparticles with Aluminum Phthalocyanine Chloride as a Nanocarrier for Modulating Immunogenic Cell Death in Murine Melanoma In Vitro
Source: Pharmaceutics. 2024 Jul 14;16(7):941. doi: 10.3390/pharmaceutics16070941 (PMC11280393; doi:10.3390/pharmaceutics16070941)
Supplement: Supplementary file 1 [file pharmaceutics-16-00941-s001.zip › pharmaceutics-3043643-supplementary.pdf]

Supplementary Material

The potential of Photodynamic Therapy associated with the nanocarrier SLN-AlPc in modulating immunogenic cell death in murine melanoma in vitro

Marina Mesquita Simões[1], Karen Letycia Rodrigues Paiva[1], Isadora Florêncio de Souza[1], Victor Carlos Mello[1], Ingrid Gracielly Martins da Silva[1], Paulo Eduardo Narcizo Souza[2], Luis Alexandre Muehlmann[3], Sônia Nair Báo[1*].

[1]Laboratory of Microscopy and Microanalysis, Department of Cell Biology, Institute of Biological Sciences, University of Brasília, Brasília 70910-900, DF, Brazil

[2]Optical Spectroscopy Laboratory, Institute of Physics, University of Brasilia, Brasília 70910-900, DF, Brazil

[3]Laboratory of Nanoscience and Immunology, Faculty of Ceilandia, University of Brasilia, Brasilia/DF, Brazil.

* Correspondence: snbao@unb.br

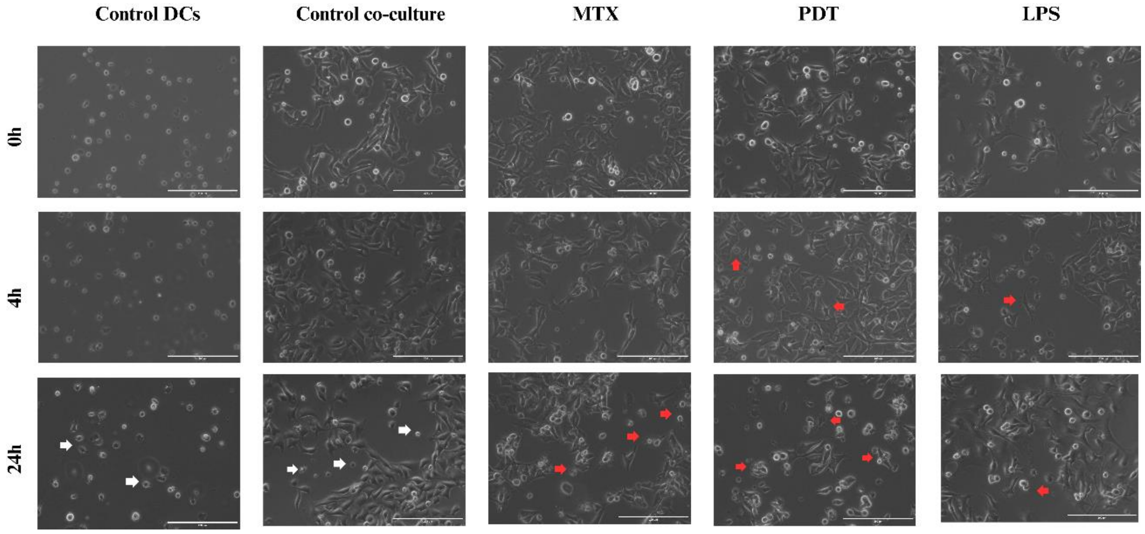

Figure S1. Light Microscopy of B16-F10 cells after treatments co-cultured with DCs. Observation of dendritic cell (DC) alterations co-cultured with B16-F10 cells treated with Photodynamic Therapy (PDT), Mitoxantrone (MTX), lipopolysaccharide (LPS) at 0, 4, and 24 hours after the start of co-culture. The morphologies of DCs alone and DCs co-cultured with untreated B16-F10 cells were also analyzed. White arrows indicate immature dendritic cells with a spherical shape, while red arrows indicate dendritic cells with altered morphology, showing dendrite elongation and interaction with B16-F10 cells.

Videos

Video S1. The 3D reconstruction video of control of HMGB1. - **https://drive.google.com/file/d/1t3JJjAGTORCE7Li_HNssI6zFi67OfsVF/view?usp=sharing**

Video S2. The 3D reconstruction video of PDT of HMGB1. - **https://drive.google.com/file/d/1NWo67zcM5Y-RaWvhq4E0m3gDPIr-YFjS/view?usp=drive_link**

Video S3. The 3D reconstruction video of MTX of HMGB1. - **https://drive.google.com/file/d/18gJQ4QAFDBH-x6r4y-xqUp034jB60DH7/view?usp=drive_link**

Video S4. The 3D reconstruction video of control of calreticulin. - **https://drive.google.com/file/d/1APUQS3QcVHF7b8wxniXzOucCi0f50WMr/view?usp=sharing**

Video S5. The 3D reconstruction video of PDT of calreticulin. - **https://drive.google.com/file/d/1bxOUAyEtEnFY-CHhysDz-tKnx-LP0x0C/view?usp=sharing**

Video S6. The 3D reconstruction video of MTX of calreticulin. - **https://drive.google.com/file/d/1m7-wCiZLURtBTqHcDlG0R5segZezLKiN/view?usp=sharing**
